# TECHNOLOGY REDUCES DISTRESS IN A GERIATRIC COHORT WITH DEPRESSION AND NEUROCOGNITIVE IMPAIRMENTS

**DOI:** 10.1093/geroni/igz038.1862

**Published:** 2019-11-08

**Authors:** James A Mazzone, Jonathan Sills, Flora Ma, Peter Louras, Erickson Alexander

**Affiliations:** 1 Veterans Affairs Palo Alto Health Care System, Menlo Park, California, United States; 2 Veterans Affairs Palo Alto Health Care System, Palo Alto, California, United States; 3 Palo Alto University, Palo Alto, California, United States

## Abstract

Many older adult Veterans with Depression and superimposed Neurocognitive Impairments may demonstrate behavioral agitation. To buffer patient agitation seen within a Veteran’s Affairs residential extended care facility, psychological services were expanded to include the use of mobile technologies. To evaluate the effectiveness of adding technology supported psychological services, outcomes were gathered as part of continuous process improvement efforts. 28 Veterans with Depression and NCI who received technology enhanced services were rated by staff on observed agitation behaviors prior and following clinical encounters. In addition, a subset of 17 Veterans also provided subjective unit of distress (SUD’s) ratings based on the Brief Interview for Mental Status inclusion criteria. Paired sample T-Tests were completed to assess if technology-enhanced interventions resulted in average reductions of clinician rated observations of Veteran agitation behaviors. Significant differences were found in observations of Veteran facial tension (t(27)=3.722, p=.001), agitated body movement (t(14)=2.020, p=.053), and threatening posture (t(27)=2.243, p=.044). Evaluation of a subset of those residents who also provided SUD’s ratings show a significant change in self-reported distress after intervention (t(16)=4.3, p=.001). Effect size for this difference was large (d=1.042). These results suggest that mobile technologies can help reduce agitation and Veteran self-reported distress among older Veterans with Depression and superimposed Neurocognitive Impairments.

